# Synanthropic rodents as virus reservoirs and transmitters

**DOI:** 10.1590/0037-8682-0486-2019

**Published:** 2020-02-07

**Authors:** Mara Lucia Gravinatti, Carla Meneguin Barbosa, Rodrigo Martins Soares, Fábio Gregori

**Affiliations:** 1 Departamento de Medicina Veterinária Preventiva e Saúde Animal, Faculdade de Medicina Veterinária, Universidade de São Paulo, São Paulo, SP, Brazil.; 2 Instituto de Ciências Biomédicas, Universidade de São Paulo, São Paulo, SP, Brazil.

**Keywords:** Viruses, One health, Urban environment, Rat

## Abstract

This review focuses on reports of hepatitis E virus, hantavirus, rotavirus, coronavirus, and arenavirus in synanthropic rodents (*Rattus rattus*, *Rattus norvegicus*, and *Mus musculus*) within urban environments. Despite their potential impact on human health, relatively few studies have addressed the monitoring of these viruses in rodents. Comprehensive control and preventive activities should include actions such as the elimination or reduction of rat and mouse populations, sanitary education, reduction of shelters for the animals, and restriction of the access of rodents to residences, water, and food supplies.

## INTRODUCTION

Rodents (Order: Rodentia) are distributed on all continents except for Antarctica^1^. Their heterogeneous and cosmopolitan distribution expands as their interaction with humans increases^2^. Some species are better able to adapt to urban environments (synanthropism).

In a study conducted in Buenos Aires (Argentina), black rats (*Rattus rattus*) were found in residential and industrial areas, while house mice (*Mus musculus*) and brown rats (*Rattus norvegicus*) were captured in green areas and shantytowns^3^, where their presence was favored by easy access, availability of shelter, and large food supply^4^.

These animals are natural reservoirs of infectious diseases^5^ ^,^ ^6^, and are involved in the emergence and dissemination of viruses, bacteria, and protozoa. The transmission of these agents can occur through both direct (bite, contact) and indirect (urine, feces) means, through vectors (ticks, fleas, and mites) that infest rodents, or when they are predated by other species^1^ ^,^ ^7^ ^,^ ^8^.

Although little information is currently available on the size of the population of synanthropic rats, some indicators allow the subjective estimation of their presence, such as the presence of excrement, marks on walls, trails on the ground, food sources, or the visual observation of rodents and/or the damage caused by them^9^ ^-^ ^11^.

To evaluate the size of rodent populations, the concept of the ‘minimum known number’ is used^12^, where individuals have to be captured and recaptured to estimate the infestation rate. This statistical formula can be simplified by multiplying the number of animals caught in a trap in a single catch by 100^11^ ^,^ ^13^.

A survey carried out in 1529 dwellings in a low-income region of São Paulo (Brazil) showed an initial synanthropic rodent infestation rate of 40%, which was reduced to 14.4% after the implementation of sanitary education and pest control^13^. Similarly, in Pau de Lima (Bahia, Brazil), 62% of households (137/221) presented signs of active rodent infestations^14^.

Besides synanthropic species, other rodents can cocirculate in rural environments and wild and urban interface areas, where they contact other animals and people^10^ ^,^ ^15^. Wild rodents are reservoirs for hantavirus, vaccinia virus, and *Lassa virus*, among others^16^ ^-^ ^19^.

Fernandes et al. (2019)^20^ investigated rural settlements in Goiás (Brazil), and found 2.57% (n=12) positive rate for *Orthohantavirus*, equally distributed between women and men (n=6). In contrast, similar studies showed higher frequency in middle-aged men due to their risk behaviors^21^ ^,^ ^22^.

Refugee camps accommodate large number of people and consequently there is accumulation of food and residues, attracting rodents (synanthropic and wild). In Africa, studies have revealed the circulation of *Mastomys natalensis* infected by *Lassa virus* in these camps, which is facilitated by the dissemination routes of the virus (urine, fomites, consumption of rodents as food)^23^. Bonner et al. (2007)^24^ investigated communities of up to 9,000 people and determined that the quality of housing, external hygiene, and the visualization of rodent burrows were the main epidemiological factors associated with the spread of *Lassa virus*.

Aircrafts and ships may also contribute to the introduction of rodents and even dissemination of diseases in new areas^25^ ^,^ ^26^. Consequently, the World Health Organization (WHO) has implemented rodent control measures at airports and ports, including periodic surveys to verify the absence of rodents onboard vessels. According to reports, 24.7% (270/1093) of moored ships at the port of Shanwei (China) were infested with rats^25^. In Heilongjian, another Chinese port area, 4.47% of the 649 collected rats tested positive for hantavirus^27^.

Therefore, this article aims to review important viral agents disseminated by synanthropic rodents (*M. musculus*, *R. rattus*, and *R. norvegicus*), and thereby contribute to a better understanding of disease epidemiology and prevention.

## SEARCH STRATEGY AND SELECTION CRITERIA

Scientific texts in English and Portuguese were retrieved from the PubMed, Scopus, Web of Science, and Scielo research platforms using the search term “virus” in combination with “disease” and “rat or rodent or murine”. Additionally, a second more refined research was performed with the terms “Hepatitis E,” “Hantavirus,” “Rotavirus,” “Coronavirus”, or “Arenavirus” associated with “rat or rodent or murine.”

Among the resulting, studies mainly related to synanthropic rodents (*M. musculus*; *R. rattus*; *R. norvegicus*) collected in the field were selected, excluding the studies restricted to animal experimentation.

Thus, hepatitis E, hantavirus, rotavirus, coronavirus, and arenavirus are the focus of this review, as they are neglected diseases transmitted by rodents.

## HEPATITIS E (HEV)

The hepatitis E virus (HEV) has a single-stranded RNA genome of approximately 7 kb length^28^. As a member of the *Hepeviridae* family, the genus *Orthohepevirus* has four species (from *A* to *D*)^29^, among which *Orthohepevirus A* and *C* have already been described in rodents^30^.

Infection occurs via the fecal-oral route through the consumption of water contaminated with excrement or the consumption of raw/undercooked meat and the viscera of infected animals^31^. The prevalence rate in humans reaches 40% in industrialized countries, and the virus has been detected in blood banks^32^. Socially vulnerable people may be an important epidemiological group at risk, along with patients who depend on blood transfusions^33^ ^,^ ^34^.

The virus shows tropism to digestive system and is eliminated in stool after 4 to 23 days of infection^35^, remaining in this state for additional 5 weeks^36^ ^,^ ^37^.

The symptoms are mainly nonspecific, and include fever, headaches, abdominal pain, but these infections may also be asymptomatic (depending on the HEV dose to which the patient was exposed), hindering the detection, and potentiating the agent’s spread^38^ ^-^ ^40^.

Mortality rates are higher among infected people presenting previous liver disease, immunocompromised patients, and pregnant women, as they present higher chances of renal failure leading to death^33^ ^,^ ^41^ ^,^ ^42^.

The role of black rats (*R. rattus*) and brown rats (*R. norvegicus*) as reservoirs and transmitters of this viral agent and its prevalence rate remains unknown^30^.

*Orthohepevirus A* has seven different genotypes (HEV1-7) defined by the concatenated amino acid distance between the open reading frames of ORF1 (nonstructural proteins) and ORF2 (capsid proteins)^43^. HEV-1 and HEV-2 occur only in humans; HEV-3 has been isolated from humans and several animal species; HEV-4 has been isolated from humans and pigs; HEV-5 and HEV-6 have been identified only in wild boars; and HEV-7 has only been found in camels^44^.

The presence of anti-HEV IgG^30^ ^,^ ^45^, and the detection of viral particles in the feces of these rodents (with or without seroconversion) have been demonstrated^37^ ^,^ ^46^ ^,^ ^47^. However, only a single study has shown similarities between rodent (*R. norvegicus*) strains with regard to the HEV-3 genotype, which is most closely related to the genotypes found in rabbits^37^.

On the other hand, *rat* HEV (genotype C1), belonging to the *Orthohepevirus C* group, has been reported in *R. norvegicus* and *R. rattus*, although its potential to cause disease in humans is still questioned^37^ ^,^ ^47^.

In Vietnam, animals captured at bus stations and hospitals have tested positive for *rat* HEV^48^. These findings are supported by serological evidence from domestic animals and rodents in other studies^45^ ^,^ ^49^ ^,^ ^50^.

Detection methods for HEV include serological (specific IgG), histopathological, and molecular (RT-PCR) techniques^41^. Commercially, there are no specific prophylatic measures available on the market, although Chinese researchers have developed the HEV p239 vaccine from the HEV1 genotype^51^. Its efficacy is considered high (> 90%), requiring three doses (0,.1 and 6 months), and it may be used even in pregnant women^52^.

## HANTAVIRUS (HV)

Hantaviruses, belonging to the *Hantaviridae* family, are divided into four genera: *Loanvirus*, *Mobatvirus*, *Thottimvirus*, and *Orthohantavirus* ^53^. These enveloped viruses have a negative-sense RNA genome segmented into three fragments: Large - L (6.8-12 kb), Middle - M (3.2-4.9 kb), and Small - S (1-3 kb). They encode four proteins; the L segment encodes viral polymerase, while the M and S segments encode the precursor (GPC) of two viral surface glycoproteins (G1 and G2, alternatively called Gn and Gc), and the nucleocapsid (N) protein, respectively^54^.

More than 50 species of hantaviruses have been reported worldwide^55^; however, some of them do not cause diseases, including the *Prospect Hill* virus^56^. Rodents, bats, and moles are reservoirs of these agents^57^; transmission occurs through bites (saliva), and especially via the inhalation of viral particles from the feces and urine of these animals^58^. Despite a report of transmission between humans, this form is rare^59^.

The *Orthohantavirus* genus includes the greatest number of pathogenic species of public health importance^60^. Its presence is associated with the geographic distribution of rodents (*Murinae, Avicolinae, and Sigmodontinae families), which can harbor distinct forms of the disease.*

The earliest reports of hemorrhagic fever with renal syndrome (HFRS), caused by viruses known as Old World hantaviruses (Europe and Asia), come from Chinese writings dating from 960 BC. Later, in the Korean War (1951-1954), these diseases caused the death of over 3,000 soldiers^61^. The *Hantaan* virus (HTNV) species was related to this outbreak. HTNV was isolated for the first time in 1978^62^ and was linked to the rodent *Apodemus agrarius*; the virus was detected in blood, urine, feces, and respiratory tract samples. In humans, this species causes a severe form of HFRS, which has thus far been restricted to rural areas of China, Korea, and Russia^63^ ^,^ ^64^.

*Dobrava* virus (DOBV) is also associated with HFRS syndrome, for which men are accidental hosts^65^. Mortality rates vary according to the genotype, ranging from 0.5% (DOBV - *Kurkino*) to 12% (DOBV - *Dobrava* and DOBV - *Sochi*)^66^ ^,^ ^67^.

The most commonly detected viral agent of this group in Western Europe, *Puumala* virus (PUUV), is disseminated by *Myodes glareolus,* whose proliferation is favored by the underbrush vegetation of this region, and the spread of the virus is further affected by virus-host coevolution^68^. In humans, it causes moderate nephropathy, and can lead to subclinical infections^69^.

*Seoul* virus usually causes mild infections and without medical care the mortality rates reach 1%. The host of this virus (*R. norvegicus*) is found in urban areas, leading to a cosmopolitan distribution of the disease, in contrast to those caused by other Old World hantaviruses^60^ ^,^ ^70^. The incubation period varies from 2-3 weeks, and the endothelial cell tropism of the virus^71^ produces nonspecific symptomatology (fever, headaches, muscle pain, nausea, vomiting), in addition to respiratory problems, dizziness, and diarrhea. Thrombocytopenia entails the development of petechias, and decreased blood pressure affects kidney function, causing renal failure followed by disseminated intravascular coagulation^72^.

Hantavirus cardiopulmonary syndrome (HCPS) is another pathology associated with agents of the genus New World hantaviruses (Americas), and appears to be related to climatic phenomena such as *El Niño* ^58^.

The first report of hantavirus in Brazil dates from 1993, when *Juquitiba* virus, transmitted by *Oligoryzomys nigripes,* was detected by Silva et al. (1997)^73^ *.* To date, the following viruses have been identified in the country: (a) *Araraquara* virus (transmitted by *Necromys lasiurus* rodents*);* (b) *Castelo dos Sonhos* virus (*Oligoryzomys utiaritensis*); (c) *Laguna Negra* virus (*Calomys callidus*); (d) *Anajatuba* virus (*Oligoryzomys mattogrossae*); and *Rio Mamore* virus (*Oligoryzomys microtis*), among others^20^.

The incubation period of HCPS ranges between 16-24 days, with initial nonspecific HFRS-like symptomatology differentiated by pulmonary edema and lymphoid organ impairment^74^, which may cause cardiovascular shock and death^72^. Studies indicate that this pulmonary phase lasts approximately 1 week, but long-lasting sequelae have been reported, such as dyspnea and weakness^61^. According to available data from the Brazilian Ministry of Health, 2061 people had been infected in the country as of 2017, with a lethality rate of 40.1%^75^.

In rodents, this virus causes a chronic infection with mild symptomatology such as decreased growth^76^ and renal problems^77^, although it is usually asymptomatic. This can be explained by the coevolution of rodents with the virus over millions of years^78^. Hantavirus can be diagnosed by associating the patient’s history with the presence of wild or synanthropic rodents. Tests for hantavirus include specific serological detection using ELISA (IgM or IgG) or viral detection using RT-PCR or real-time PCR^79^.

The occurrence of HFRS has not yet been reported in Brazil, despite serologically positive human and rodent samples^80^. For example, in an urban area in Salvador, Brazil, *Seoul* virus antibodies were found in *R. norvegicus* serum samples^81^. A molecular survey conducted in Madagascar detected the *Anjozorobe* virus (Thailand *Orthohantavirus*) strain in *R. rattus* and *M. musculus*, suggesting viral spillover^82^.

To our knowledge, there is no licensed vaccine available on the market that prevents hantavirus infections. Several clinical trials at different stages are ongoing to test inactivated (monovalent and bivalent), DNA, and live attenuated vaccines for both HFRS and HCPS, with effectiveness of approximately 93.77-100% being reported^83^-^85^. Additionally, studies have demonstrated that the use of antiviral ribavirin increases the survival rate in hantavirus-infected rats^86^ ^-^ ^88^.

## ROTAVIRUS (RV)

Rotavirus (RV) belongs to the *Rotavirus* genus within the *Sedoreovirinae* subfamily of the *Reoviridae* family^53^. These nonenveloped viruses have a double-stranded RNA genome of approximately 18550 bp in length, fragmented into 11 segments. The genome encodes six structural (VP1, VP2, VP3, VP4, VP6, and VP7) and six nonstructural (NSP1, NSP2, NSP3, NSP4, and NSP5/6) proteins, as the NSP5/6 gene is bicistronic^89^ ^,^ ^90^.

Myriad mechanisms of viral variability occur in RVs, such as point mutations, rearrangements, reassortments, and intragenic recombination, conferring great genetic diversity^91^ ^,^ ^92^. This genus is divided into nine different groups (RVA-RVI) based on the antigenic properties and nucleotide sequences of the VP6 protein^89^ ^,^ ^93^ ^,^ ^94^, and there is a potential candidate tenth group, RVJ^95^.

For the RVA group, it is necessary to adopt the notation of Gx-P[x]-Ix-Rx-Cx-Mx-Ax-Nx-Tx-Ex-Hx, which considers all the variability presented by the coding genes of the VP7-VP4-VP1-VP2-VP3-NSP1-NSP2-NSP3-NSP4-NSP5/6 proteins, respectively^96^. To date, this group has at least 36 known G genotypes and 51 known P genotypes in humans and animals^93^ ^,^ ^97^ ^-^ ^99^.

Although rotaviruses are considered species-specific, heterologous infections may occur^100^. Wa-like and DS1-like (RVA) strains primarily cause disease in humans, and infections caused by genotypes and serotypes common to animals have also been documented^100^ ^,^ ^101^.

Synanthropic rodents are usually not associated with RVs, however, their efficiency in disease transmission and their close contact with other animals and people highlight their importance to the epidemiology of these viruses^101^ ^,^ ^102^.

Transmission initially occurs through the fecal-oral route, via particles present in the soil and water, causing diarrhea due to the loss of absorptive capacity of injured intestinal cells during viral replication^103^. Diarrhea is a leading cause of infant mortality worldwide, and rotavirus infections are responsible for more than 35% of these cases^104^.

RVs are widely distributed in Brazil, and have been described in animals (both young and adults) such as cattle^105^, birds^106^, and pigs^107^ ^,^ ^108^. In rodents, there is a single report of RVA associated with swine production^109^.

A metagenomic analysis of *R. norvegicus* in Germany characterized a sample of RVA, revealing close identity between the identified strain and other animal and human strains, namely, genotypes G3, P[3], and N2^110^.

In Italy, 40 fecal samples from *R. rattus* collected on swine farms were analyzed, and a sample of RVA was characterized (G3-P[3]-I1-R11-C11-M10-A22-N18-T14-E18-H13), demonstrating an atypical combination of genotypes^102^.

As the associated symptomatology is mainly nonspecific, the diagnosis can easily be misleading^111^. Commercial ELISA kits, RT-PCR (single or multiplex), and qPCR assays^99^ ^,^ ^102^ ^,^ ^110^ are available for the detection of these infections.

To control this disease, animal vaccination (swine and cattle) should be carried out, mainly in females in the late gestation period. In production animals, prevalence rates may be higher than 90% in adults^103^. For humans, two vaccines are authorized by the WHO: (a) Rotarix® (GlaxoSmithKline Biologicals, Rixensart, Belgium, an attenuated strain of G1P [8] RVA) and (b) RotaTeq® (Merck & Co., Whitehouse Station, NJ, with five strains of genotypes G1P [5], G2P [5], G3P [5], G4P [5] and G6P [8]).

## CORONAVIRUS (COV)

 Coronaviruses are enveloped, nonsegmented, positive, single-stranded RNA viruses associated with the structural N-phosphoprotein in a nucleocapsid with helical symmetry^112^ ^,^ ^113^. They are found in a wide variety of animals causing respiratory, enteric, hepatic, and neurological diseases of varying severity^114^. According to the International Committee on Taxonomy of Viruses, two subfamilies belong to the *Coronaviridae* family; *Letovirinae*, which has one subgenus, *Milecovirus*, found only in frogs and a sea hare thus far^115^, and *Orthocoronavirinae*, which is found in birds and mammals, and is divided into four genera due to the antigenic and genetic characteristics of the viruses^53^ ^,^ ^116^.

Phylogenetic studies indicate that bats are the gene source for *Alpha* and *Betacoronaviruses,* while birds are the gene source for *Gama and Deltacoronaviruses* ^117^. Thus, *Alpha* and *Betacoronaviruses* are found mainly in mammals, such as humans, dogs, cats, pigs, bats, mice, rats, horses, and cattle^114^ ^,^ ^118^ ^-^ ^123^, while *Gama* and *Deltacoronaviruses* infect mainly birds, with exceptions such as the white whale *Gamacoronavirus* (*Delphinapterus leucas*)^124^ and the porcine *Deltacoronavirus* ^125^.

Among the *Alpha* and *Betacoronaviruses*, six are of public health importance, causing mild (HCoV-229E, NL63, OC43, and HKV1) to severe respiratory syndromes (SARS and MERS)^120^ ^,^ ^126^ ^,^ ^127^.

Despite the many uncertainties about the epidemiology and reservoirs of severe acute respiratory syndrome (SARS) and Middle Eastern respiratory syndrome (MERS), bats have been identified as the most likely reservoirs, while palm civets (*Paguma larvata*)^128^ and dromedary camels (*Camelus dromedarius*)^129^ ^,^ ^130^ act as intermediary hosts before dissemination to humans^120^ ^,^ ^131^ ^,^ ^132^. Both diseases have caused worldwide health problems, affecting 27 countries and causing hundreds of deaths in 2002 (SARS) and 2012 (MERS), aggravated by nosocomial transmission or transmission by family members^126^.

In general, the virion contains at least three proteins: the spike (S), membrane (M), and envelope (E) proteins. In addition, some coronaviruses include hemagglutinin esterase (HE)^133^. Proteins M and E are related to viral assembly^134^, while the S protein show hemagglutinating activity and is the main target for neutralizing antibodies^135^.

The S protein, which shows great variability, is responsible for host specificity because its S1 and S2 subunits are used for binding the virus to host cell receptors, and are associated with the antigenicity and pathogenicity of the virus^112^ ^,^ ^136^.

Many similarities exist between the CoVs of rats and bats^119^ ^,^ ^120^ ^,^ ^140^ ^,^ ^141^, suggesting that rodents could act as important reservoirs^142^. A survey conducted on 330 intestinal content samples from rodents (*Apodemus sp*., *Myodes glareolus*, *Arvicola terrestris*, and *Microtus sp*.), collected between 2014 and 2016 in different regions of France^121^, revealed positivity of 6.3% (21 samples), all belonging to *Alphacoronavirus* groups. This study also revealed *Alpha* and *Betacoronavirus* in bats, rabbits, and hedgehogs from the same area^121^.

An investigation conducted in China^120^, analyzed 177 rodent intestinal samples from three different species (*Apodemus chevrieri*, *Apodemus ilex*, and *Eothenomys fidelis*), and found *Alpha* and *Betacoronavirus* in 13% (23 samples). 

Besides field investigations, rodent CoV also has an important role as Murine hepatitis virus (MHV)^137^, and has been used for experimental infections, mostly for the identification of potential viruses showing interspecies transmission^119^. Although there is little information about the prevalence and diversity CoV in rodents^138^ ^,^ ^139^, many new species of *Alpha* and *Betacoronaviruses* (LRNV, LAMV, LRLV, and HKU24) have been identified in rodents in China and Europe^119^ ^,^ ^120^ ^,^ ^140^ ^,^ ^141^.

 Some vaccine candidates are being developed only for MERS-CoV, including whole-virus, vectored virus, DNA, and protein-based vaccines, however, lack of investment is delaying their development^143^.

## ARENAVIRUS (AV)

Arenaviruses (genus *Arenavirus*, family *Arenaviridae*) are enveloped viruses with an RNA genome segmented in two ambisense single stranded molecules: small (S) and large (L)^53^. The S portion encodes nucleocapsid protein and envelope glycoproteins, while L segment contains RNA dependent RNA polymerase (RdRp) and zinc-binding protein genes. Both S and L intergenic regions may potentially form one or more hairpins, which regulate mRNA transcription^53^ ^,^ ^144^ ^-^ ^146^. 

This viral family has four genera, based on phylogenetic analysis involving pairwise sequence comparisons (PASC) of complete genomes^53^ ^,^ ^147^. *Antennavirus* genus includes viruses that infect frogfish^148^, *Hartmanivirus* and *Reptarenavirus* infect snakes, and *Mammarenavirus*, which have been reported in bats, ticks, rodents, and primates, including humans^146^ ^,^ ^147^. 

*Mammarenavirus*es are correlated to the geographical location where their hosts are found^147^; currently classified as: (a) Old World (OW) (*Lassa* virus, *Lujo* virus, among others), and found mainly in Africa in rodents of the *Murinae* family as natural hosts^149^. Although *Lymphocytic choriomeningitis* virus (LCMV) belongs to this category, it circulates globally^146^. Approximately 5% of the human population have been exposed to LCMV, due to the ubiquity of the virus host, *M. musculus* ^150^; (b) New World (NW), found in the American continent, are divided into four clades (A-D)^151^. Examples of NW viruses are *Junin* virus - Argentina^152^, *Machupo* virus - Bolívia^153^, *Sabiá* virus - Brazil^154^, and *Guanarito* virus - Venezuela^155^. *Sigmodontinae* rodents are the main hosts of this class, even though *Tacaribe* virus has already been described in bats^156^ and *Amblyomma americanum* ticks^157^.

Members of both OW and NW arenaviruses can cause hemorrhagic fever and severe human diseases affecting the central nervous system^158^. Zoonotic transmission is through contact with rodents’ urine or feces, and human-to-human transmission is possible^146^. Because of their impact on human health and rapid spread, they are potential bioterrorism agents^159^.

In Colombia, a study conducted with *M. musculus*, collected from residential areas, detected 10% (8/80) of positives in serological analysis for LCMV. When brain samples of the same animals were submitted to RT-PCR, serologically negative individuals showed positive results in this second analysis, highlighting the importance of parallel diagnosis^160^. It can be justified by the vertical transmission among rodents that may deactivate cytotoxic T- lymphocytes, generating immune-complexes that may lead to misdiagnosis of ELISA reactions^160^.

A research conducted in French Guiana sampled 37 animals (*M. musculus*) of which two were positive for LCMV by hemi-nested PCR (from lung and kidney samples)^161^; another inquiry in Argentina reported that 9.4% of the mice collected were positive for arenavirus, and the serological rate was 4.6% and 2.6% for men and women, respectively^162^. In Baltimore (USA), 9% of the mice were seropositive for LCMV^163^, and 4.7% of the people were analyzed^164^. In Brazil, to our knowledge, there are no serological records on the prevalence of LCMV in rodents or humans.

*Lassa* virus (OW) is endemic in African countries, with seroprevalence reaching about 50% of the human population; this disease causes about 5,000 deaths every year^165^. *Mastomys natalensis* is considered the main reservoir of the virus^166^, but it can also be found in *Hylomyscus pamﬁ* and *Mastomys erythroleucus* ^167^.

Surveys conducted in Nigeria reported positive animals for *Lassa virus* in *M. natalensis* and *M. erythroleucus*, *R. rattus* ^168^, and *M. musculus* ^169^. In China, RT-PCRs performed in organs of *R. rattus* and *R. norvegicus* showed positive rates of 75% and 17%, respectively, and a new viral species, the *Wenzhou* virus, was isolated^145^.

*Junin* virus is considered endemic in Argentina^170^ and sporadic outbreaks have been reported^171^. There are several promising vaccinal prototypes being developed for this virus, most are in the preclinical stage^172^ and one, based on plasmidial DNA, has already reached human test phase^173^.

In Brazil, the most remarkable arenavirus is *Sabiá* virus, reported in São Paulo (Brazil) in 1994. Initial symptoms were described as flu-like (fever, sickness, headaches, and lethargy), quickly leading to hemorrhage and death (within 3 days)^154^ ^,^ ^174^. There have been two reports of this virus, caused by occupational exposure in a laboratory environment, one in Pará (Brazil) and the other in Connecticut (USA), both with non-fatal courses^175^ ^,^ ^176^. The 4th case was described in São Paulo (Brazil) in 1999^177^ and the 5th, on January 2020 in Sorocaba (São Paulo, Brazil)^178^ as a natural infection with lethal outcome.

A new arenavirus, namely *Pinhal* virus, has been characterized as a New World arenavirus (line C), first isolated from vesper mice (*Calomys tener*) in São Paulo (Brazil), but there is still no evidence that this viral strain causes disease to humans^177^ ^,^ ^179^. Besides *Pinhal* virus, other arenaviruses have been reported in Brazil: *Xapuri* virus was recently isolated from rodents (*Neacomys musseri*); *Amaparí* virus (*Neacomys guianae*); *Cupixi* virus (*Oryzomys megacephalus*); *Flexal* virus (unidentified *Oryzomyini* rodent); *Oliveros* virus (*Necromys lasiurus*); *Latino* virus (*Calomys callosus* and *Calomys callidus*) and *Aporé* virus (*Oligoryzomys mattogrossae*)^180^.

Arenaviruses can be diagnosed using: (a) RT-PCR (fluids, feces, and tissues) followed by viral RNA sequencing for differentiation; (b) serology, through detection of specific IgG and IgM employing immunofluorescence and/or ELISA tests; (c) viral isolation in cell culture.

Recommended treatment is support therapy that can be combined with the antiviral ribavirin, which should be administered during the first 7-10 days after infection. Despite its efficacy, there are significant side effects, such as hemolytic anemia, progressive weight loss, respiratory difficulty, insomnia, and dermatitis, among others^180^ ^-^ ^182^. Alternative drugs with less side effects have been tested, such as favipiravir^183^ and triarylmethane clotrimazole^184^. Cocktails using multiple antiviral drugs that target different steps of the viral life cycle appear to be the best strategy to limit viral multiplication with lower risk of drug resistance^185^.

In literature we find description of viral rodent-infections, usually from within the context of biological models or experimentation. In this review, we focus on rodents within an urban environment, especially *R. rattus*, *R. norvegicus*, and *M. musculus*, although with the advancement of human populations, the interaction with wild rodents increases, and different viruses can emerge. There are relatively few studies addressing the monitoring of viruses in these hosts, favoring the occurrence of outbreaks.

Control and preventive activities should go beyond the elimination or reduction of the populations of these hosts and involve sanitary education to aid the human population in the reduction of shelters for the hosts, the restriction of rodent access to residences, and the reduction of their water and food supply. Basic sanitation actions are a generic but effective measure in the reduction of rodents and, consequently, the propagation of diseases.
