# Erratum

**DOI:** 10.1590/0037-8682-0486b-2019

**Published:** 2020-06-08

**Revista da Sociedade Brasileira de Medicina Tropical/Journal of the Brazilian Society of Tropical Medicine**

**Title:** Synanthropic rodents as virus reservoirs and transmitters

Vol.53:e20190486: 2020 - Page: 4/11 - doi: 10.1590/0037-8682-0486-2019

Here is the form in which the information is found: “According to the International Committee on Taxonomy of Viruses, two subfamilies belong to the *Coronaviridae* family; *Letovirinae*, which has one subgenus, *Milecovirus*, found only in frogs and a sea hare thus far^115^, and *Orthocoronavirinae*, which is found in birds and mammals, and is divided into four genera due to the antigenic and genetic characteristics of the viruses^53,116^.”

It should be read: “According to the International Committee on Taxonomy of Viruses, two subfamilies belong to the *Coronaviridae* family; *Letovirinae*, which has one subgenus, *Milecovirus*, found only in frogs (Microhyla fissipes) thus far^115^, and *Orthocoronavirinae*, which is found in birds and mammals, and is divided into four genera due to the antigenic and genetic characteristics of the viruses^53,116^”

